# Chemokine (C-X-C) ligand 1 (CXCL1) protein expression is increased in aggressive bladder cancers

**DOI:** 10.1186/1471-2407-13-322

**Published:** 2013-07-01

**Authors:** Makito Miyake, Adrienne Lawton, Steve Goodison, Virginia Urquidi, Evan Gomes-Giacoia, Ge Zhang, Shanti Ross, Jeongsoon Kim, Charles J Rosser

**Affiliations:** 1 Cancer Research Institute, Orlando Health, Orlando, FL 32827, USA; 2 Department of Pathology, Orlando Health, Orlando, FL 32806, USA; 3 Nonagen Bioscience Corporation, Orlando, FL 32827, USA

**Keywords:** Bladder Cancer, Chemokine Ligand 1 (CXCL1), Tumor Grade, Tumor Stage

## Abstract

**Background:**

Chemokines, including chemokine (C-X-C motif) ligand 1 (CXCL1), may regulate tumor epithelial-stromal interactions that facilitate tumor growth and invasion. Studies have linked CXCL1 expression to gastric, colon and skin cancers, but limited studies to date have described CXCL1 protein expression in human bladder cancer (BCa).

**Methods:**

CXCL1 protein expression was examined in 152 bladder tissue specimens (142 BCa) by immunohistochemical staining. The expression of CXCL1 was scored by assigning a combined score based on the proportion of cells staining and intensity of staining. CXCL1 expression patterns were correlated with clinicopathological features and follow-up data.

**Results:**

CXCL1 protein expression was present in cancerous tissues, but was entirely absent in benign tissue. CXCL1 combined immunostaining score was significantly higher in high-grade tumors relative to low-grade tumors (*p* = 0.012). Similarly, CXCL1 combined immunostaining score was higher in high stage tumors (T2-T4) than in low stage tumors (Ta-T1) (*p* < 0.0001). An increase in the combined immunostaining score of CXCL1 was also associated with reduced disease-specific survival.

**Conclusion:**

To date, this is the largest study describing increased CXCL1 protein expression in more aggressive phenotypes in human BCa. Further studies are warranted to define the role CXCL1 plays in bladder carcinogenesis and progression.

## Background

Chemokines are known to be critical mediators of the inflammatory response by regulating the recruitment of immune cells from both the innate and adaptive immune systems to diseased tissues. Dysregulated expression and activity of certain chemokines has been implicated in the initiation and progression of several cancers. Specifically, the chronic exposure of cells to a chemokine-rich milieu is associated with macrophage and T cell accumulation, chronic activation of macrophages, abnormal angiogenesis, and DNA damage due to the presence of reactive oxygen species [1,2]. Furthermore, chemokines have been known to regulate multiple processes associated with tumor progression including primary tumor growth, tumor angiogenesis and development of metastatic disease, thus some reports have linked chemokines to more aggressive cancers [3,4]. One chemokine of interest is chemokine (C-X-C) ligand 1 (CXCL1), also known as growth-regulated oncogene-alpha or melanoma growth stimulatory activity, alpha. CXCL1 has been reported to be overexpressed in colon, skin and breast cancers [5-8], but only one study to date has reported on the expression pattern of CXCL1 protein in human bladder tumors. Herein, we report the largest study assessing CXCL1 expression patterns in human bladder cancer (BCa) tissues.

## Methods

Under MD Anderson Cancer Center Orlando/Orlando Health Institutional Review Board approval with waiver of informed consent, 142 bladder tumor paraffin blocks and 10 benign bladder paraffin blocks dating from 2002–2009 were identified in the Department of Pathology archives at Orlando Health, Inc. The clinicopathologic variables of the study cohort are shown in Table 1. All paraffin blocks were examined by H&E for histological verification of disease status.

**Table 1:** Demographic and clinicopathologic characteristics of 152 subjects comprising IHC study cohort

	**IHC cohort**	
	**BCa (%) n = 142**	**Controls (%) n = 10**
**Median age (range, y)**	73 (30–94)	29 (15–48)
**Median age (range, y)**	73 (30–94)	29 (15–48)
**Race**		
**Caucasian**	134 (94%)	N/A
**African American**	5 (4%)	N/A
**Other**	3 (2%)	N/A
**Median follow-up (months)**	13	11
**Clinical stage**		
**Tis**	16 (11%)	
**Ta**	6 (4%)	
**T1**	60 (42%)	
**≥T2**	60 (42%)	
**Tumor grade**		
**Low**	11 (8%)	
**High**	131 (92%)	

Abbreviations: *IHC* immunohistochemistry, *BCa* bladder cancer, *N/A* not available.

### Immunohistochemistry staining

Paraffin blocks were cut (5 μm sections) and placed on a Superfrost Plus Microslide. Sections were deparaffinized followed by antigen retrieval using citric acid buffer (pH 6.0, 95°C for 20 minutes). Slides were treated with 1% hydrogen peroxide in methanol to block endogenous peroxidase activity. After 20 minutes of blocking in 1% bovine serum albumin (BSA), the slides were incubated overnight at 4°C with anti-human CXCL1 antibody (sc-1374; goat polyclonal, dilution 1/250 in 1% BSA) from Santa Cruz Biotechnology (Santa Cruz, CA). Next, the slides were incubated with 2 μg/mL of biotinylated anti-goat IgG secondary antibody (Vector Laboratories, Burlingame, CA) for 30 minutes at room temperature. Subsequently, the sections were stained using Standard Ultra-Sensitive ABC Peroxidase Staining kit (Pierce/Thermo Fisher Scientific, San Jose, CA) and 3, 3′- diaminobenzidine (DAB; Vector Laboratories), counterstained by hematoxyline, dehydrated, and mounted with a cover slide. Mouse xenograft tumors from the human prostate cancer cell line PC-3, known to stain strongly for CXCL1 [9] were used as a positive control.

### Quantification of CXCL1 expression of bladder cancer

Using light microscopy, two investigators (MM and AL), who were blinded to patients’ data, interpreted immunostaining results. The sections were analyzed and staining assessed using a semiquantitative grading system based on a previous report by Eck *et al*. [10]. Briefly, the location of immunoreactivity (*e*.*g*., nuclear, cytoplasm, cell membrane, and stroma) was noted. The expression level of CXCL1 was scored by assigning a proportion score and an intensity score. The proportion of CXCL1-positive cells was scored in four grades and represented the estimated proportion of immunoractive cells (0 = 0% of cells; 1 = 1% to 40%; 2 = 41% to 75% and 3 = 76% to 100%). The intensity was scored and represented the average intensity of positive cells (0 = none; 1 = weak; 2 = intermediate and 3 = strong). The proportion and intensity scores were added to obtain a combined immunostaining score for the expression of CXCL1, which ranged from 0 to 6 (0, none; 1–2, low; 3–4, moderate; and 5–6, high). A third investigator (CJR) reviewed discrepancies and rendered a final score.

### Statistical analyses

Correlation between the pathologic variables and CXCL1 levels was analyzed using Chi-square test. Disease-specific survival (DSS) and overall survival (OS) curves were obtained using the Kaplan-Meier method, and compared by the log-rank test for each prognostic variable. Variables with effect on survival in univariate analysis were included in the Cox proportional hazard regression model. Multivariate analysis was performed to identify independent prognostic variables using a stepwise Cox proportional hazards regression model. IBM SPSS Version 21 (SPSS Inc., Chicago, IL) and PRISM software version 5.00 (San Diego, CA) were utilized for statistical analyses and plotting the data, respectively. Statistical significance in this study was set at *p* < 0.05 and all reported *p* values were 2-sided.

## Results

**Figure 1:**
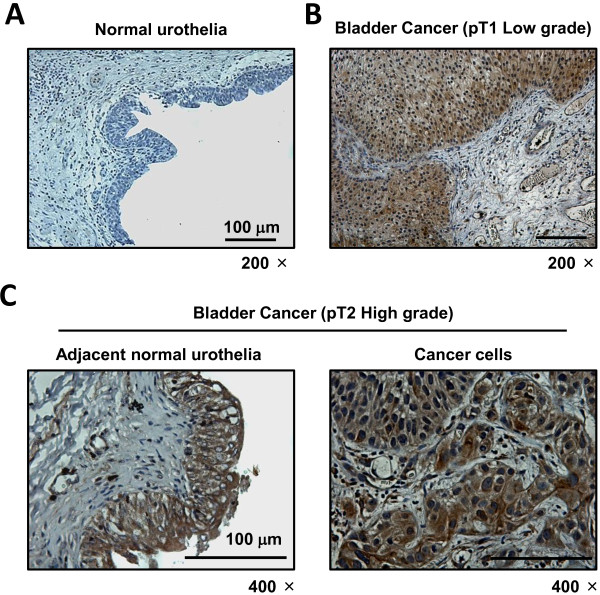
**Expression of CXCL1 protein in human bladder.** Representative staining of benign bladder tissue **A**, bladder cancer **B**, and tumor-adjacent normal urothelia within section with cancer **C**. All images were captured at 200× or 400× magnification.

We set out to analyze CXCL1 protein levels in benign and cancerous bladder tissue samples using immunohistochemical staining. In benign bladder tissues, CXCL1 expression was entirely absent (Figure 1A). Conversely, in tumor tissue, varying CXCL1 expression was found in both epithelial and stromal components of the tissue (Figure 1B). Within the stroma, CXCL1 immunoreactivity was diffuse and was not associated with any specific cell types. Within the epithelia, CXCL1 immunoreactivity was localized to the cytoplasm. In many cases we were able to compare cancerous epithelia cells and tumor-adjacent, histologically benign epithelial cells within the same tissue section. Interestingly, tumor-adjacent urothelia also expressed high levels of CXCL1 (Figure 1C), suggesting that in urothelial components, early molecular changes occur well before morphological changes become evident. This finding supports the ‘field-effect’ phenomenon that has been used to describe a change in the entire urothelium once a carcinoma lesion is established [11].

**Figure 2:**
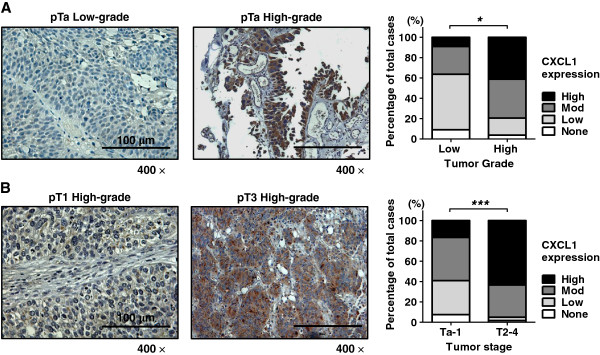
**Expression of CXCL1 protein in high**-**grade and high stage human bladder cancer. A**, Representative staining of low-grade bladder tumor and high-grade bladder tumor. Bar graph illustrates combined immunostaining score for CXCL1 expression according to tumor grade. **B**, Representative staining of non-muscle invasive bladder cancer (pT1) and muscle invasive bladder cancer (pT3). Bar graph illustrates combined immunostaining score for CXCL1 expression according to tumor stage. All images were captured at 400× magnification. *, *p* < 0.01 and ***, *p* < 0.0001.

Based on the combined immunostaining score, CXCL1 protein expression was significantly different between low-grade and high-grade tumors (*p* = 0.01). Some 7% of low-grade tumors exhibited ‘high’ staining, whereas 46% of high-grade tumors had ‘high’ staining (Figure 2A). Similarly, higher stage tumors (*e*.*g*., MIBC stages T2-T4) had significantly higher CXCL1 expression (*p* < 0.0001) than lower stage counterparts (*e*.*g*., NMIBC stages Ta-T1). For example, 63% of T2-T4 tumors had ‘high’ CXCL1 staining, compared to only 18% of Ta-T1 tumors (Figure 2B).

**Figure 3:**
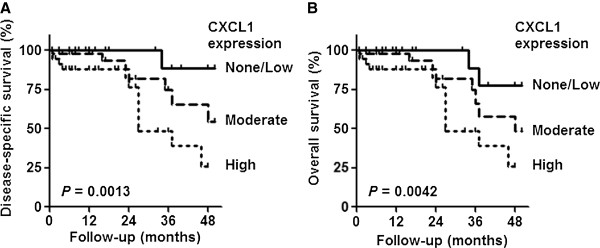
**Probability of bladder cancer specific survival according to CXCL1 staining patterns.** ‘High’ combined immunostaing score for CXCL1 was associated with a significant reduction in **(A)** disease-specific survival and **(B)** overall survival (Log-rank test).

Due to reduce numbers of cancer samples with ‘none’ or ‘low’ combined immunostaining score, these two groups were combined for subsequent survival analysis. Univariate Cox analysis revealed that increased CXCL1 expression represents a poor prognostic factor regarding DSS (Figure 3A) and OS (Figure 3B) (*p* = 0.0013 and *p* = 0.0042, respectively). Reduced DSS and OS were also associated with MIBC (*p* < 0.0001 and *p* = 0.0015, respectively) (Table 2). MIBC was also an independent risk factor for DSS and OS (*p* = 0.001 and *p* = 0.001, respectively) in multivariate analyses (Table 3).

**Table 2:** Univariate analysis of disease specific survival and overall survival

**Variables**			**Disease**-**specific survival**			**Overall survival**		
		**N**	**HR**	**95% CI**	***p***	**HR**	**95% CI**	***p***
**Age (yrs)**								
	≤ 65	35	1			1		
	> 65	107	1.88	0.69–5.08	0.21	2.03	0.78–5.25	0.15
**Sex**								
	Male	115	1			1		
	Female	27	1.45	0.47–4.46	0.52	1.3	0.44–3.86	0.63
**Tumor grade**								
	Low	11	1			1		
	High	131	2.97	0.42–20.90	0.27	2.99	0.46–19.58	0.25
**Stage**								
	NMIBC	82	1			1		
	MIBC	60	11.78	4.71–29.43	<0.0001	8.83	3.65–21.32	0.0015
**Combined immunostaining score for CXCL1**								
	None / Low	34	1			1		
	Moderate	53	3.45	0.84–14.13	0.085	2.68	0.76–9.50	0.13
	High	55	5.73	1.92–17.10	0.0018	4.63	1.60–13.37	0.0047

Abbreviations: *HR* Hazard ratio, *95% CI* 95% confidence interval, *NMIBC* Non-muscle invasive bladder cancer, *MIBC* Muscle invasive bladder cancer.

**Table 3:** Multivariate analysis of disease specific survival and overall survival

**Variables**	**Disease**-**specific survival**					**Overall survival**		
		**N**	**HR**	**95% CI**	***p***	**HR**	**95% CI**	***p***
**Stage**								
	NMIBC	82	1			1		
	MIBC	60	13.61	2.79–66.39	0.001	7.63	2.25–25.92	0.001
**Combined immunostaining score for CXCL1**								
	None / Low	34	1			1		
	Moderate	53	1.57	0.17–14.58	0.69	1.17	0.21–6.36	0.86
	High	55	3.05	0.34–27.16	0.32	2.08	0.39–10.98	0.39

*HR* Hazard ratio, *95% CI* 95% confidence interval, *NMIBC* Non-muscle invasive bladder cancer, *MIBC* Muscle invasive bladder cancer.

## Discussion

With an estimated 70,980 newly diagnosed cases of BCa and 14,330 deaths in 2012, cancer of the urinary bladder is the second most common genitourinary malignancy in the U.S. and among the five most common malignancies worldwide [12]. Urothelial carcinoma, the most prevalent histologic subtype, accounts for 90% of all BCa in the US [13]. More than 70% of newly diagnosed BCa are NMIBC at first presentation and treatment can be curative with transurethral resection with or without adjuvant intravesical therapy. NMIBC possesses a high recurrence rate (~70%) but a low progression rate (~15%) and an excellent 5-year survival rate of 94% [14]. Some thirty percent of patients are diagnosed with MIBC or advanced BCa. MIBC cases are usually treated by radical cystectomy or external beam radiation therapy with concomitant chemotherapy. The 5-year survival in these patients does not exceed 50% [15]. Our understanding regarding the carcinogenesis and progression of BCa is still limited. Better insight into the molecular background of BCa is needed in hopes of improving outcomes. Reports have linked BCa initiation to chronic inflammation [16,17], and the dysregulated expression and activity of certain chemokines has been implicated in the potential progression from an inflammatory environment to cancer initiation [3,4]. This chronic inflammatory milieu is associated with macrophage and T cell accumulation, chronic activation of macrophages, abnormal angiogenesis, and DNA damage due to the presence of reactive oxygen species [1,2].

CXCL1 has been reported to be overexpressed in gastric, colon, skin and renal cancers [5-8,18]. However, its presence has also been negatively associated with non-small cell lung cancers [19], attesting to its complex role in tumorigenesis and angiogenesis. CXCL1 protein expression had not previously been intensely investigated in BCa, but it is functionally closely related to interleukin 8 (IL-8), which has been widely studied in BCa [20-22].

One other study supports the association of CXCL1 in more aggressive BCa. Based on proteomic profiling of bladder cancer cell lines, Kawanishi *et al*. monitored urinary CXCL1 obtained from 67 patients with BCa [23]. The study showed that urinary CXCL1 levels were significantly higher in patients with MIBC relative to NMIBC (*p* = 0.0028), and that CXCL1 was an independent factor for predicting the invasive phenotype.

**Figure 4:**
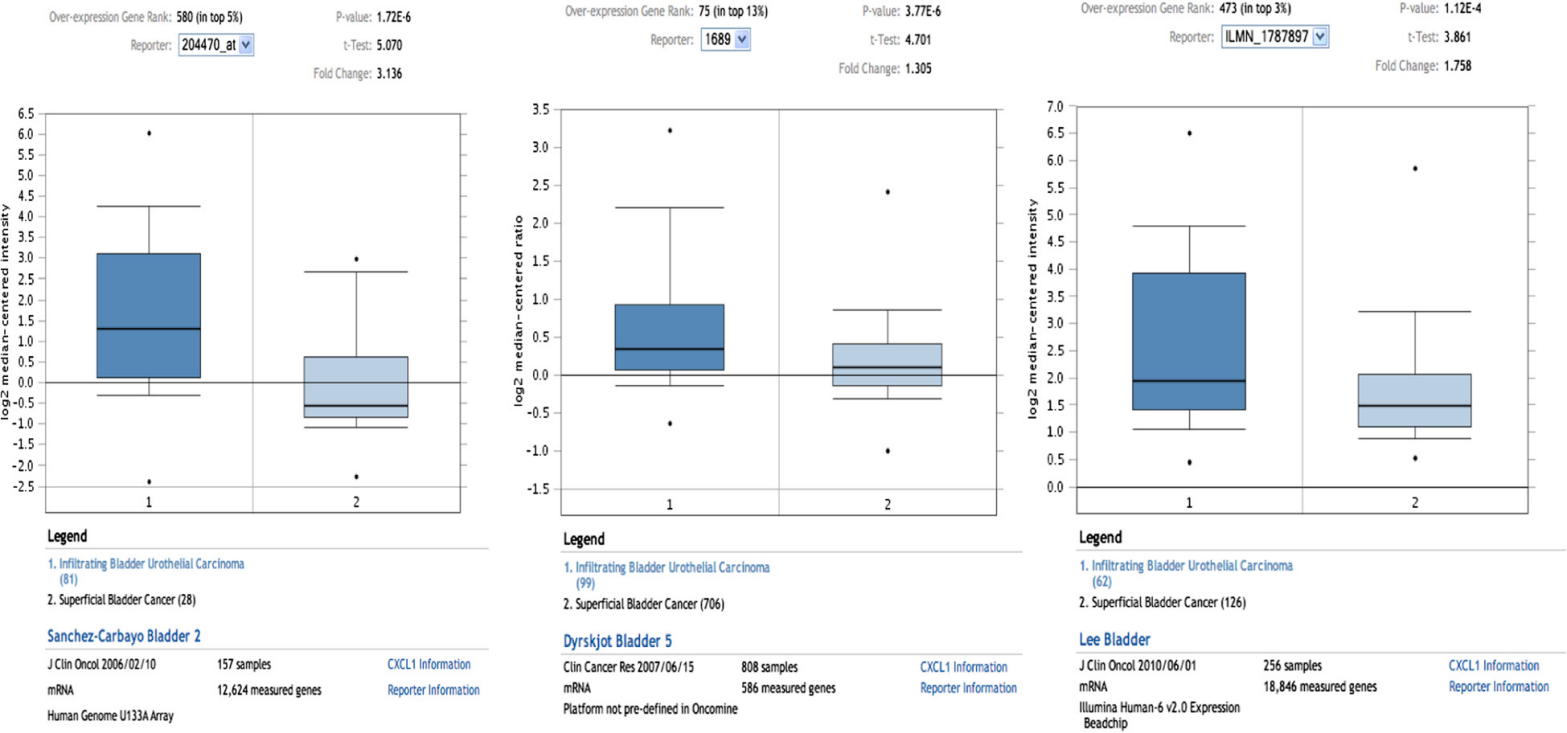
**CXCL1 mRNA expression in human bladder cancer from publically available datasets.** Three public datasets containing transcriptome profiles of bladder tumor tissues were obtained and analyzed for CXCL1 expression using Oncomine 3.0. Mean relative mRNA levels for CXCL1 in muscle invasive bladder cancer cases was significantly elevated compared to non-muscle invasive bladder cancer cases (Sanchez-Carbayo *et al*., *p* = 1.72^-6^[28], Dyrskjot *et al*. *p* = 3.77^-6^[29], and Lee *et al*., *p* = 1.12^-4^[30]).

We report the first large-scale study assessing CXCL1 protein expression in human bladder tumor tissues. Increased expression of CXCL1 was evident in higher grade and higher stage bladder tumors. Furthermore, higher CXCL1 levels were associated with a reduction in disease-specific survival. Similar survival results have been reported in gastric cancer [24], breast cancer [25] and melanoma [26,27]. These findings support a role for CXCL1 in bladder tumor initiation and progression, and further studies are underway to better define the influence of CXCL1 in BCa. While no previous CXCL1 protein studies had been reported in bladder tumors, a number of studies have profiled the BCa transcriptome. In order to investigate the specific pattern of CXCL1 mRNA expression in bladder tissue we analyzed three publicly available datasets [28-30] using Oncomine 3.0 [31]. Our data-mining analyses illustrated CXCL1 expression in BCa tissues, and revealed a significant elevation of CXCL1 in MIBC compared to NMIBC (Figure 4), further substantiating our findings that CXCL1 was noted to be significantly different between NMIBC *vs*. MIBC. Data related to tumor grade were not available for analysis.

Our study has several limitations. Though we are able to demonstrate a significant difference in the protein expression of CXCL1 in benign tissues compared to cancerous tissues, our benign cohort was quite small (n = 10). Furthermore, we had a limited range of low-grade tumors (n = 11) thus hampering detailed statistical analysis of our cohort. Lastly, follow-up data of our BCa subjects was relatively short (median follow-up 13 months), which limits long term prognostication. Additional studies are underway taking these limitations into consideration.

## Conclusions

Our study demonstrated that CXCL1 protein is specifically expressed in human bladder carcinomas and that increased expression is associated with high-grade and high stage BCa, and with reduced DSS. Further studies may provide insight into the molecular mechanisms associated with a proinflammatory microenvironment that may contribute to cancer development and progression. Knowledge of the role of CXCL1 in tumor progression may facilitate the design of new therapeutic approaches that inhibit tumor cell growth through selective perturbation of CXCL1 function.

## Abbreviations

CXCL1: Chemokine (C-X-C) ligand 1; BCa: Bladder cancer; HR: Hazard rate; CI: Confidence interval; NMIBC: Non-muscle invasive bladder cancer; MIBC: Muscle invasive bladder cancer.

## Competing interests

The following authors declare that they have no competing interests. Makito Miyake, Adrienne Lawton, Ge Zhang, Shanti Ross, Jeongsoon Kim, Evan Gomes Giacoia.

The following authors declare that they have a competing interest. Steve Goodison – affiliated with Nonagen Bioscience Corp., Virginia Urquidi – affiliated with Nonagen Bioscience Corp., Charles J Rosser – affiliated with Nonagen Bioscience Corp.

## Authors’ contribution

MM carried out IHC staining, reviewed IHC staining, performed statistical analysis and drafted M&M section of the manuscript. AL reviewed IHC staining. GZ, JK and EGG carried out IHC staining. SR collected and organized clinical data. SG participated in study design and helped to draft the manuscript. VU collected and organize array data present in Figure 4. CJR conceived the study, and participated in its design and coordination and helped to draft the manuscript. All authors read and approved the final manuscript.

## Pre-publication history

The pre-publication history for this paper can be accessed here:

http://www.biomedcentral.com/1471-2407/13/322/prepub
